# Primary Amenorrhea in an 18-Year-Old Phenotypic Female With a 46,XY Karyotype: Complete Androgen Insensitivity Syndrome

**DOI:** 10.7759/cureus.100864

**Published:** 2026-01-05

**Authors:** Radhika Katiyar, Dileep K Chaurasia, Arun Kumar, Shirish Mishra, Archit Sabberwal

**Affiliations:** 1 General Surgery, Moti Lal Nehru Medical College, Prayagraj, IND; 2 Urology, Moti Lal Nehru Medical College, Prayagraj, IND

**Keywords:** amenorrhea, androgen-insensitivity syndrome, bilateral orchidectomy, chromosome karyotype, intra-abdominal testes

## Abstract

Complete androgen insensitivity syndrome (CAIS) is an uncommon disorder of sexual differentiation in which a genetically male (46,XY) individual presents with a typical female phenotype due to androgen receptor resistance. We report the case of an 18-year-old phenotypic female with primary amenorrhea and lower abdominal pain. Clinical assessment showed normal breast development with markedly reduced body and pubic hair. Hormonal studies revealed elevated LH and testosterone levels. Imaging confirmed the absence of Müllerian structures, and karyotyping demonstrated a 46,XY pattern. Bilateral laparoscopic orchidectomy was carried out, followed by estrogen therapy and psychological support. This case highlights the clinical clues essential for the early diagnosis of CAIS and underscores the need for empathetic counselling and coordinated multidisciplinary care.

## Introduction

Complete androgen insensitivity syndrome (CAIS) is a disorder of sexual development that is classified as a rare disease [1]. Patients are identified with chromosome 46(XY) with testes as their gonads. The estimated incidence of CAIS ranges from 1 in 20,000 to 1 in 99,000 genetically male births. These patients are identified as females phenotypically, as their bodies are completely insensitive to androgen [2]. Because these children are raised as girls and show no outward abnormalities in early development, the condition often goes unrecognized until adolescence, when primary amenorrhea prompts clinical investigation. Early identification is important, as undescended testes may carry a risk of malignant transformation later in life.

The present case report documents an 18-year-old phenotypic female who presented with primary amenorrhea and lower abdominal discomfort, ultimately diagnosed as CAIS.

## Case presentation

An 18-year-old phenotypic female presented with a 4-year history of intermittent lower abdominal discomfort and absence of menstruation. She had been raised as a female and had no past medical or surgical history. There was no known family history of disorders of sexual development, and the patient’s parents were non-consanguineous.

On examination, the abdomen was soft with no palpable mass. Per-vaginal evaluation revealed a blind vaginal pouch measuring approximately 3 cm in depth. The breasts were well-developed, corresponding to Tanner stage IV. Pubic and axillary hair were sparse, consistent with Tanner stage I. There were no features of virilization.

A detailed endocrine evaluation was performed, which is shown in Table 1. Total testosterone was measured at 18.42 nmol/L, which falls within the male reference range, suggesting the presence of functional testicular tissue.

**Table 1:** Hormonal profile of the patient AMH: anti-Mullerian hormone; TSH: thyroid-stimulating hormone; LH: luteinizing hormone; FSH: follicle-stimulating hormone

Hormonal Marker	Patient Result	Adult Female Reference (Follicular Phase)	Status (Context: 46,XY)
Serum AMH	23 ng/mL	0.9 – 9.5 ng/mL	High (typical in AIS due to the presence of Sertoli cells)
TSH	2.46	0.4 – 4.5 mIU/L	Normal
Estradiol	<10 pg/mL	30 – 400 pg/mL	Low
LH	34.06 mIU/mL	2.4 – 12.6 mIU/mL	Markedly elevated
FSH	3.19 mIU/mL	3.5 – 12.5 mIU/mL	Normal
Testosterone	18.42 nmol/L	0.3 – 2.4 nmol/L	Normal (Male range) / High (Female range)

Chromosomal analysis confirmed a 46,XY karyotype, supporting the suspicion of an androgen receptor-related disorder. Ultrasonographic examination showed non-visualization of the uterus and ovaries, with only the lower segment of the vaginal canal being identified.

**Figure 1:**
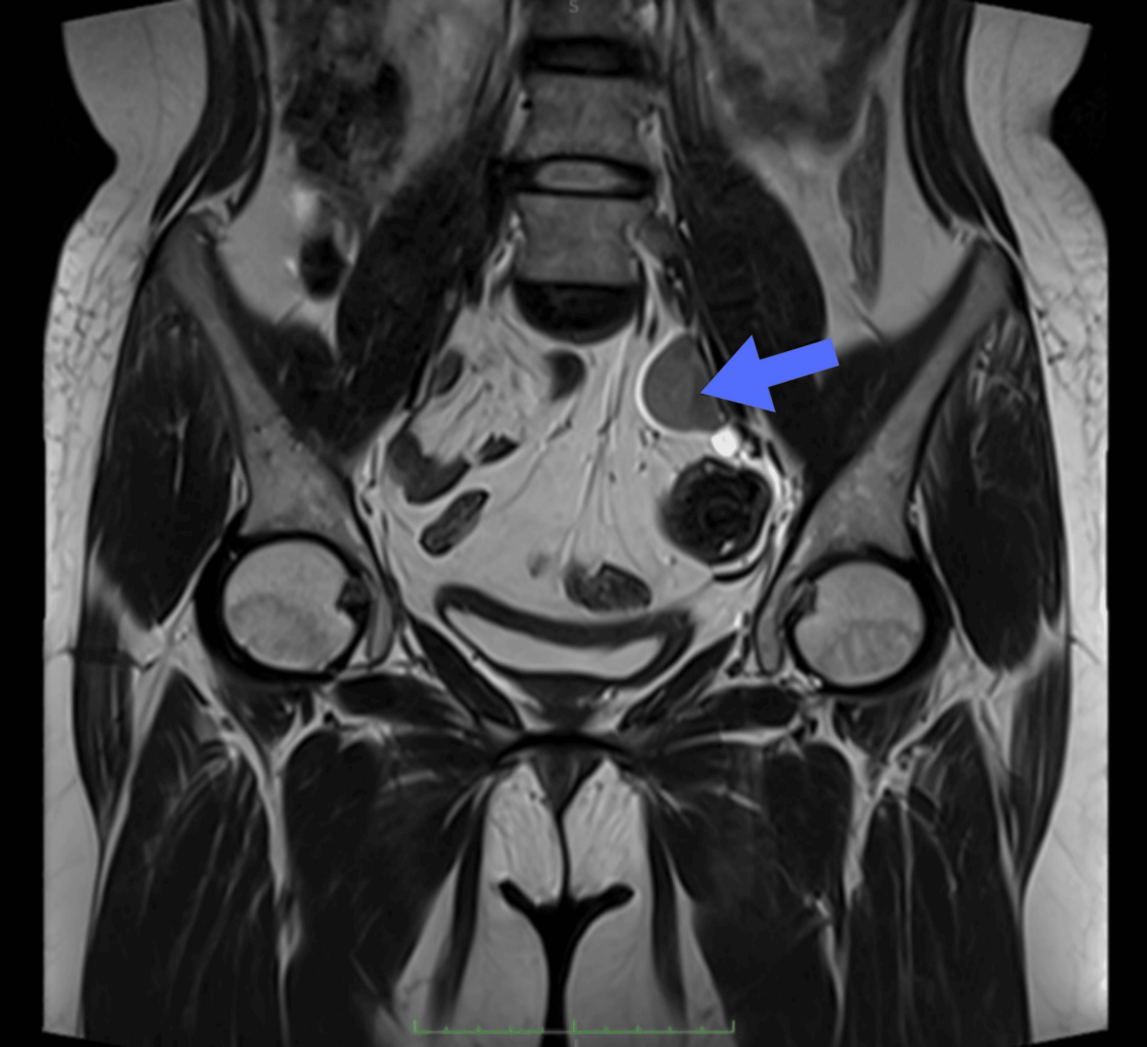
MRI pelvis scan showing well-defined ovoid tissue in the left iliac region (blue arrow)

MRI showed the absence of the uterus, cervix, and the upper two-thirds of the vagina, with a normally formed lower vaginal segment. Ovarian tissue was not seen. A 24 × 21 mm ovoid structure with small cystic components was present in the left iliac region near the external iliac vessels, suggesting testicular tissue (Figure 1). Both inguinal canals were normal. The overall findings supported a diagnosis of androgen insensitivity syndrome, with Mayer-Rokitansky-Küster-Hauser (MRKH) syndrome considered a secondary possibility.

The clinical picture in this patient, marked by normal breast maturation, very sparse pubic and axillary hair, absence of menstruation, and lack of uterine and cervical structures on imaging, together with laboratory findings of normal-to-high testosterone levels and a confirmed 46,XY karyotype, pointed clearly toward a diagnosis of CAIS. Other causes of primary amenorrhea, such as Müllerian agenesis (MRKH), gonadal dysgenesis, and hypogonadotropic hypogonadism, were initially considered; however, these were excluded based on the hormonal pattern, imaging studies, and genetic evaluation.

**Figure 2:**
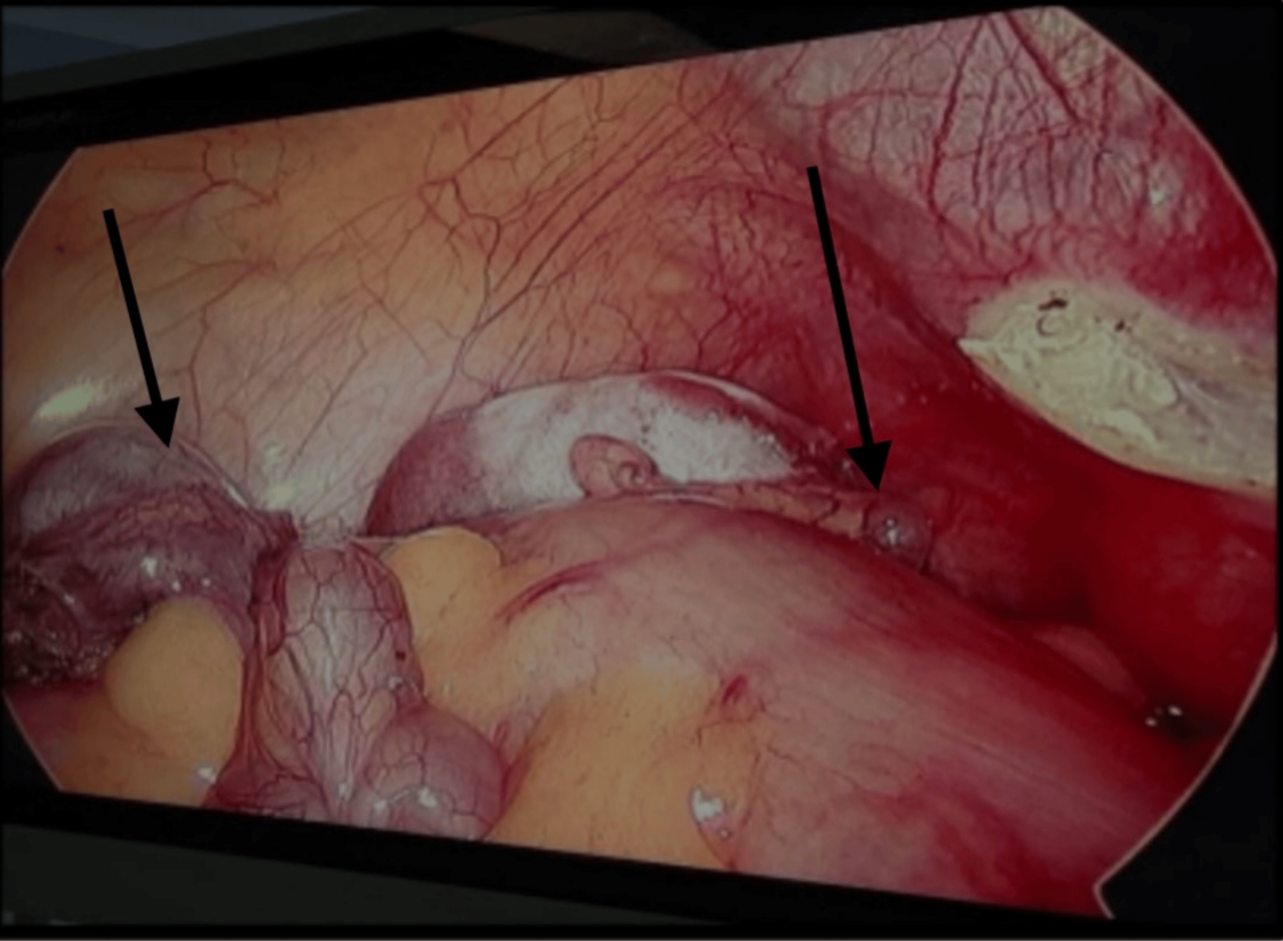
Intraoperative laparoscopic view showing bilateral intraabdominal testes (black arrows)

**Figure 3:**
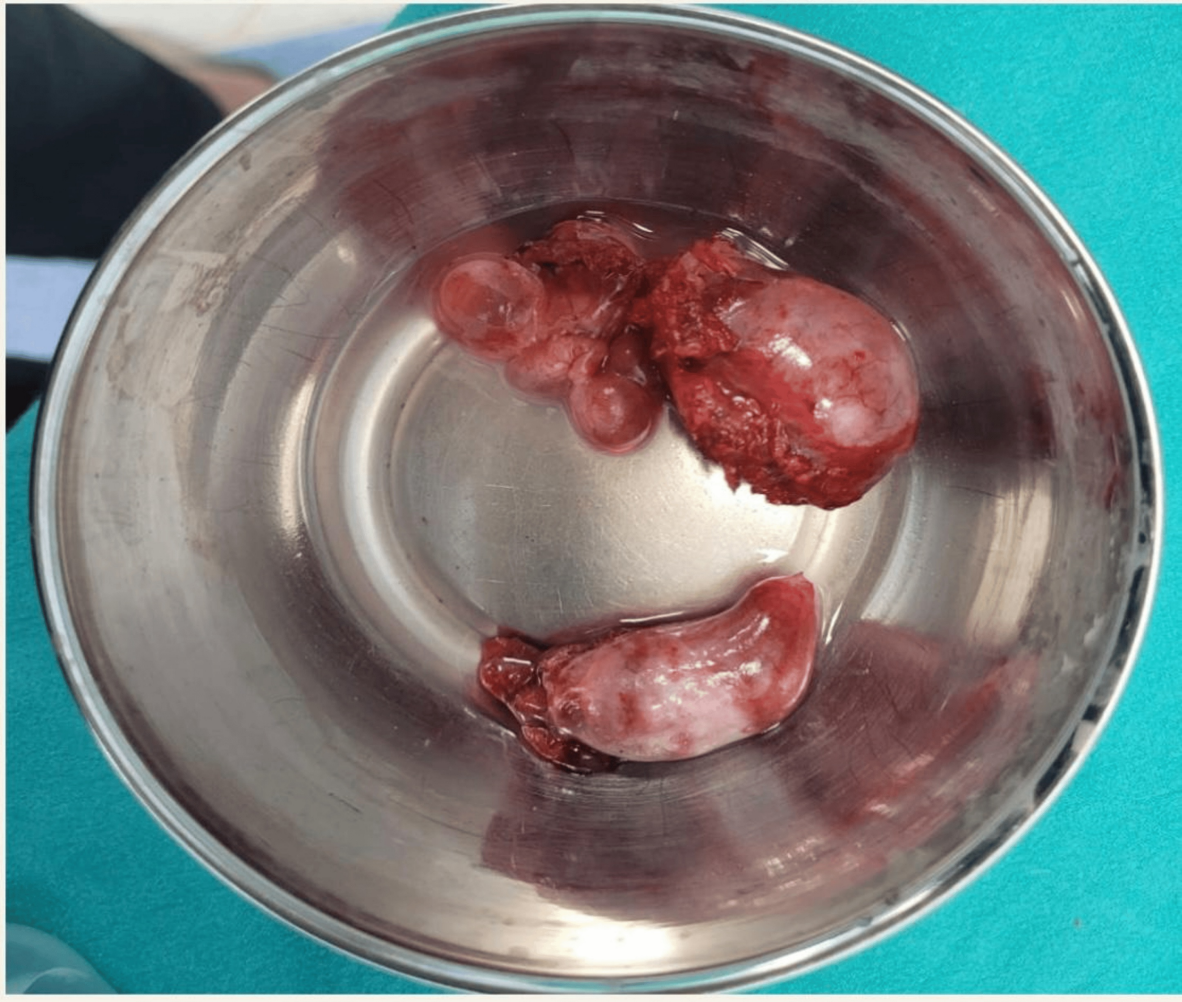
Excised testicular tissue from the pelvic cavity

After detailed counselling, bilateral laparoscopic orchidectomy (Figure 2) was performed to eliminate the potential malignancy risk associated with intra-abdominal testes. Bilateral testicular specimens, as shown in Figure 3, were removed. Postoperative tissue analysis revealed cryptorchid testicular tissue with Sertoli cell nodules, confirming retained testicular elements.

Post-surgery, the patient was commenced on lifelong estrogen replacement therapy to maintain secondary female sexual characteristics and bone health. Psychiatric counselling was provided to support emotional adjustment to the diagnosis.

Clinical outcomes and follow-up

The postoperative course was uneventful. At follow-up, the patient reported improvement in abdominal symptoms. She demonstrated good understanding of her condition and maintained stable emotional well-being with continuing psychological support and endocrine follow-up.

Patient perspective

"I always believed I was like any other girl. The diagnosis was shocking at first, but I am relieved to understand the reason behind the absence of menstruation. I continue to identify as female, and this diagnosis has not altered my gender identity. The counselling sessions have helped me accept my condition and move forward."

## Discussion

This case is consistent with CAIS, supported by clinical findings and imaging, and managed according to standard treatment protocols. CAIS results from mutations in the androgen receptor (AR) gene located at Xq11-12, which leads to a failure of androgen-mediated masculinization and consequent female phenotype in a 46,XY individual [3]. A variety of AR gene mutations have been identified, with many inherited from the maternal germline, and others arising as de novo post-zygotic mutations [4]. In our patient, a complete genetic assessment of family members was not performed and is planned for follow-up.

Patients with CAIS typically demonstrate well-developed breasts due to peripheral aromatization of excess androgen to estrogen. Their average height usually exceeds that of females but remains below the typical male range [5]. Internal Müllerian structures are absent owing to anti-Müllerian hormone secretion by the testes. Sparse axillary and pubic hair is a recognized finding reflecting androgen resistance, and cryptorchidism is generally present. Importantly, individuals with CAIS have no reproductive potential due to the absence of a functional uterus and ovaries. The malignancy risk of intra-abdominal testes gradually increases after puberty, which reinforces the indication for timely gonadectomy in these patients.

The management of CAIS includes gonadectomy followed by hormone replacement therapy (HRT). A multidisciplinary care model, encompassing surgical, gynecological, endocrinological, and psychological expertise, is essential for optimal physical and psychosocial outcomes. Pre-adult HRT promotes appropriate breast development, growth, and bone maturation [6]. In adults with CAIS post-gonadectomy, HRT aims to maintain female secondary sexual characteristics, preserve bone density, support cardiovascular health, and promote emotional well-being. Therapy is generally continued until the expected age of menopause, with transdermal estradiol currently recommended as the preferred modality [7]. In addition, counseling regarding gender identity, fertility implications, disclosure to family, and long-term psychological support is a critical component of care. For patients with a shortened vaginal canal, vaginal dilation or surgical vaginoplasty may be considered to facilitate sexual comfort and function [8]. MRKH syndrome may present similarly but is distinguished by a 46,XX karyotype with functioning ovaries, aiding differentiation from CAIS. Unlike gonadal dysgenesis, patients with CAIS exhibit normal breast development and lack streak gonads.

## Conclusions

This case highlights the clinical presentation of complete androgen insensitivity syndrome, where a mutation in the androgen receptor gene results in the inability of tissues to respond to androgens, leading to a phenotypically female presentation despite a 46,XY karyotype. Recognition is often triggered by evaluation for primary amenorrhea during adolescence, though discrepancies between karyotype and prenatal imaging may also raise suspicion earlier. This report emphasizes the importance of timely diagnosis, sensitive communication of findings, and involvement of a multidisciplinary care team, including surgery, gynecology, endocrinology, and mental health support, to guide management and counseling. Increased awareness of CAIS can help avoid delayed diagnosis and ensure appropriate counselling and timely gonadectomy.
